# Simulated warming shifts the flowering phenology and sexual reproduction of *Cardamine hirsuta* under different Planting densities

**DOI:** 10.1038/srep27835

**Published:** 2016-06-14

**Authors:** YuSong Cao, Yian Xiao, Haiqun Huang, Jiancheng Xu, Wenhai Hu, Ning Wang

**Affiliations:** 1 School of Life sciences, Jinggangshan University, Ji’an, Jiangxi Province, 343009, People’s Republic of China; 2 Key Laboratory for Biodiversity Science and Ecological Engineering, Ji’an, Jiangxi Province, 343009, People’s Republic of China

## Abstract

Climate warming can shift the reproductive phenology of plant, and hence dramatically reduced the reproductive capacity both of density-dependent and -independent plant species. But it is still unclear how climate warming affects flowering phenology and reproductive allocation of plant under different planting densities. Here, we assessed the impact of simulated warming on flowering phenology and sexual reproduction in the ephemeral herb *Cardamine hirsuta* under four densities. We found that simulated warming delayed the onset of flowering averagely for 3.6 days but preceded the end of flowering for about 1 day, which indicated climate warming shortened the duration of the flowering. And the flowering amplitude in the peak flowering day also dramatically increased in the simulated warming treatment, which caused a mass-flowering pattern. Climate warming significantly increased the weights of the fruits, seeds and seed, but reduced fruit length and sexual reproductive allocation under all the four densities. The duration of flowering was shortened and the weights of the fruits, seeds and seed, and sexual reproductive allocation were reduced under The highest density.

The relationship between the size and the density of plants is a critical focus in plant ecology. The effects of density on plant individuals and populations have been extensively investigated on such plant species as barley and wheat crops^1^,^2^,^3^, wheat^4^, *Brussels sprouts (Brassica oleracea*)^5^,^6^, *et al*. The individual growth of plant is limited by the availability of resource when plant density increased, resulting in decrease of individual plasticity in size and thus affecting plant survival, growth and reproduction. Many plant species respond to crowding by exhibiting such phenotypic plasticity as adjusting biomass allocation, and altering flowering phenology^7^. In a high-density population, plant individuals may flower early to complete their reproductive cycle before the onset of inclement conditions and maximize fecundity despite of insufficient resources^8^.

Research on plant reproductive phenology is to study regularity of such reproductive phenomenon of plant as flower bud blossoms, flowering, seed propagation, and to understand the relationships between plant reproduction and various biological and abiotic environments^9^. Flowering phenology is a critical characteristic of flowering plants^10^, and an important constituent in understanding the degree of threat to biodiversity in response to changing environmental conditions^11^. Phenological investigations at the plant population level may improve our understanding of the mechanisms of ecosystem response to climate warming^12^, which is expected to threaten greatly to global biodiversity^13^,^14^. The mean global temperature has increased by approximately 0.74 °C over the last century (IPCC, Intergovernmental Panel on Climate Change)^15^, and is predicted to rise by at least 2 °C by 2050 to 2100 (IPCC)^16^. Such climate warming may drive thousands of plant species to the brink of extinction over the next century by changing the timing of their life cycles^17^,^18^, hampering individual survival^19^, and changing species interactions^17^. However, plants may respond to climate warming by changing their phenology, distribution range, and physiology^20^. Climate warming is associated with an average advancement in the phenology of life-history traits, including migration and reproduction, in many species^19^. The rise of temperature led plants to flower early^8^. For instance, the date of onset flowering has been advanced about 4 d in temperate zones over the past century^8^. However, some phenological responses may vary among plant species in different communities and with different growth forms^12^. The duration of flowering decreases gradually with warming^12^. Some plants flower less abundantly, or even lose the opportunity to flower, when the temperature increases by more than 5 °C^21^, which indicates that climate warming may be a restraint to sexual reproduction. However, little conclusive information is currently available in this respect.

Climate change is expected to alter patterns of species co-occurrence, in both space and time^22^. Forrest reported the idiosyncratic effects of snowmelt timing on flowering duration, with some species flowering for longer periods in late years and some flowering longer in early years^22^. Because the co-occurring species can potentially attract and support the populations of interacting animals, particularly pollinators, and generalist seed predators or florivores^23^, climate shifted the flowering patterns, and thus are likely to affect plant fecundity^24^,^25^,^26^.

Thus, both population density and climate change can affect the flowering phenology and sexual reproduction of flowering plants, but the independent and interactive contributions of the two factors are poorly understood. Furthermore, the magnitude of variation in sexual reproduction of plants in response to future climate change is unknown. To explore the response of plant flowering phenology and sexual reproduction to climate warming, in the present study, we quantitatively measured phenotypic changes in flowering phenology and sexual reproductive parameters in the ephemeral herb *Cardamine hirsuta* (Brassicaceae). *C. hirsuta* is a very special plant species which can continuously grow from early the spring to the winter and produce 4 to 5 generations within a year. The specific hypotheses were that (1) climate warming would shift the flowering phenology of *C. hirsuta* and result in different under different population densities, and (2) climate warming would promote plant growth but restrain sexual reproduction, and especially decrease reproductive allocation.

## Results

### Flowering phenology

**Figure 1:**
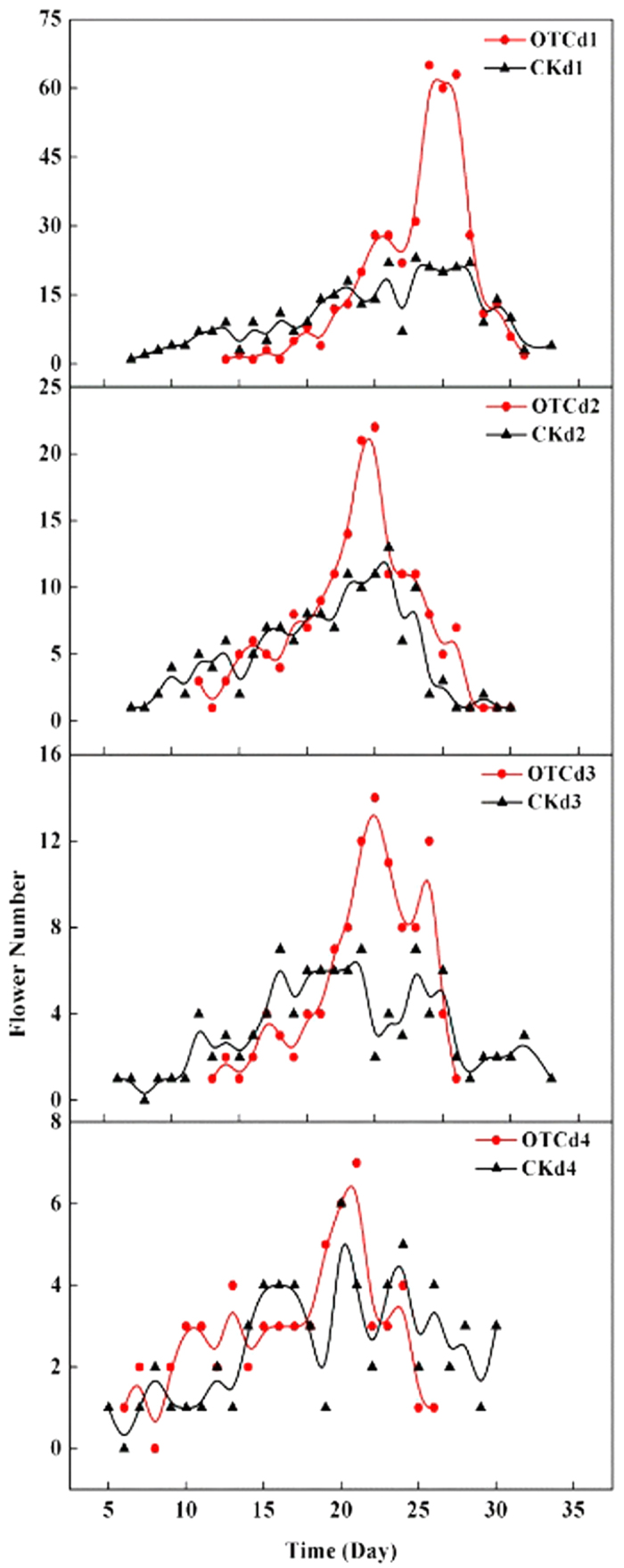
Flowering phenology curves for experimentally warmed *Cardamine hirsuta* plants grown under four planted densities. d1 to d4: 600, 2000, 4000, and 10,000 plants m^−2^, respectively; OTC, open-top chamber; CK, control.

Significant differences in the onset and duration of flowering were observed among the density treatments and the temperature treatments as well (Table 1, Fig. 1). The first flowering dates under high density were taken forward about 5 d, but the duration of flowering was shortened more than 10 d than that in CK. The first flowering dates in OTCs under different density were 4~7 days with an average 5.5 days late than that in CK, and the last flowering dates in OTCs were 1~8 days with an average 4.4 days early than that in CK (Fig. 1), which indicated the simulated warming shortened the duration of flowering about 5 d.

At the population level, significant differences in mean amplitude (flowers/plant/day), end, and duration of flowering were observed among the density treatments with warming treatments or not (Table 1, Fig. 1). However, no significant differences in the first flowering dates among the population densities were observed under simulated warming.

Significant differences in flowers quantity per plant were observed among the density treatments, and the temperature treatments as well (Table 1).

### Fruit characteristics

**Figure 2:**
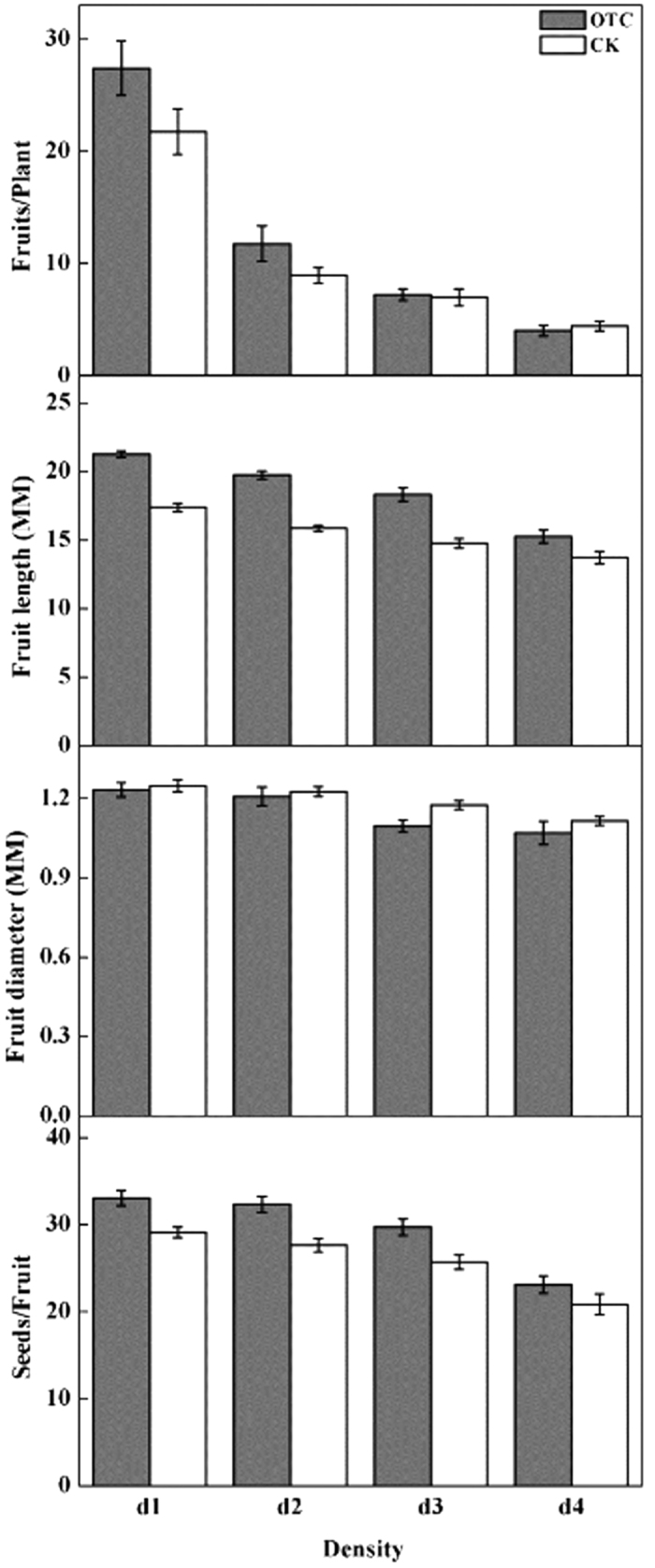
Fruiting traits of experimentally warmed *Cardamine hirsuta* plants grown under four population densities. d1 to d4: 600, 2000, 4000, and 10,000 plants m^−2^, respectively; OTC, open-top chamber; CK, control. **p < 0.01; NS, *p* > 0.05.

Both plant density and warming significantly affected the quantity of fruit per plant, fruit length, and fruit diameter (Table 1, Fig. 2). The quantity of fruit per plant, fruit length, and fruit diameter decreased significantly with increasing plant density (n = 4, P < 0.001). Compared with the CK, simulated warming in the low density treatment (d1 and d2) increased significantly the fruit quantity per plant and the fruit length, but decreased the fruit diameter.

### Seed characteristics

**Figure 3:**
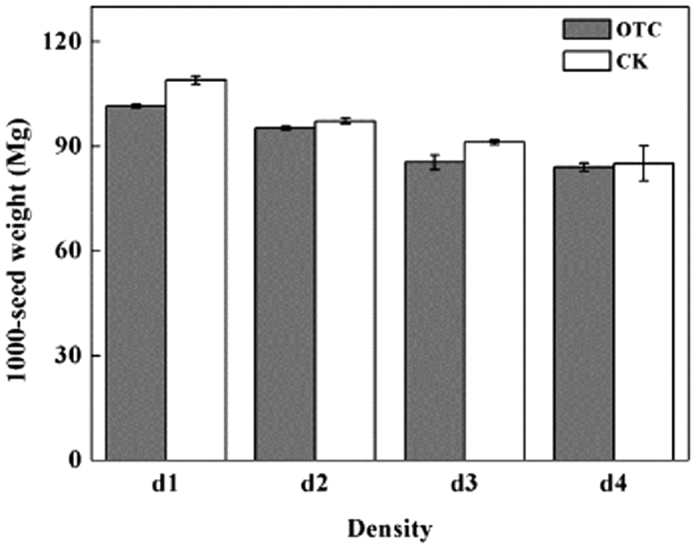
Thousand-seed weight of experimentally warmed *Cardamine hirsuta* plants grown under four population densities. d1 to d4: 600, 2000, 4000, and 10,000 plants m^−2^, respectively; OTC, open-top chamber; CK, control. *p < 0.05; **p < 0.01; NS, *p* > 0.05.

Both plant density and warming significantly affected the quantity of seeds per fruit. The quantity of seeds per fruit decreased with increasing plant density but increased with warming (Table 1, Fig. 2). Significant decreases were observed in 1000-seed weight in response to warming (Fig. 3). Furthermore, the 1000-seed weight decreased significantly with increasing plant density.

### Reproductive allocation

**Figure 4:**
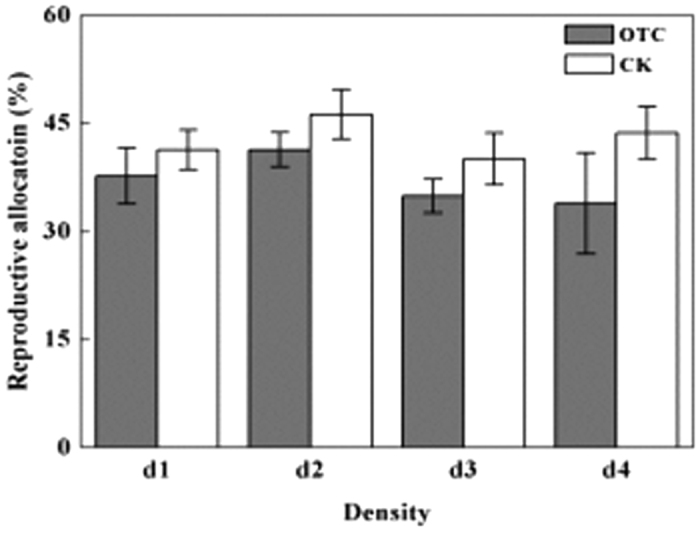
Reproductive allocation of experimentally warmed *Cardamine hirsuta* plants grown under four population densities. d1 to d4: 600, 2000, 4000, and 10,000 plants m^−2^, respectively; OTC, open-top chamber; CK, control. **p < 0.01.

Reproductive allocation (RA) was defined as weight of reproductive structures as a proportion of total above-ground biomass. The result showed that the simulated warming significantly decreased reproductive allocation under all plant densities (*F*_1,15_ = 12.706, *P* < 0.0001). However, the planted density did not significantly affect reproductive allocation, except at the highest plant density, regardless of the presence or absence of warming (Fig. 4).

## Discussion

Plants display substantial variation in flowering phenology in response to environmental changes^8^. In the present study, warming and plant density shifted the flowering phenology of *C. Hirsuta* in the population-level, which indicated that the flowering phenology of *C. hirsuta* was highly responsive to population density and climate warming. The results also showed that the initial time of flowering was taken forward but the duration of flowering was shorten with the plant density increasing, which cause the plant to flower massed in a few days. This phenomenon is defined as “mass-flowering” pattern or “cornucopia” with a well-defined peak and marked synchrony^27^. This pattern may be propitious for plant reproduction because it may attract higher numbers of pollinators or improve the effectiveness of pollination. On the contrary, warming delayed the first flowering dates and prolonged the duration of flowering, which would degrade the effectiveness of pollination.

Consistent with some previous studies^27^,^28^,^29^, the present results suggested that plants which show an early onset of flowering tend to have longer flowering durations than plants that have a later date of flowering onset. However, we observed a different pattern from the results of Buide^27^, who showed that plants which produce many flowers tend to have long flowering durations, and those of Dieringer^28^, who showed that plants which start to bloom early tend to produce higher numbers of flowers and fruit. In the present study, *C. hirsuta* showed a significantly shortened duration of flowering and higher peak flowering amplitude, and produced higher numbers of flowers and fruit in the OTCs in comparison with the ambient controls (Table 1).

Climate warming also shifts the date of flowering onset, i.e., simulated warming leads to significantly earlier onset of flowering in *Silene acaulis*^30^, *Leontodon autumnalis* var. *taraxaci*^31^, *Hibbertia hirsuta*^32^, and *Gentiana formosa*^33^. Satake, *et al*.^21^ reported that temperature increase caused *Arabidopsis halleri* to show advanced onset and shortened duration of flowering, even leading to loss of flowering opportunities in some species because of their differential responses to climate warming. Fitter and Fitter^10^ observed that 10 out of 385 British plant species show delayed onset of flowering in relation to climate warming. Liu, *et al*.^34^ observed that warming significantly delays the onset of flowering in *Aster alpinus* and *Trollius farreri* on the Tibetan plateau. In our study, *C. hirsuta* plants showed significant delays in the onset and end of flowering after warming.

It was reported^35^ that the response of species to climate warming varied significantly. For most plant species, climate warming may lead to earlier flowering in spring and delayed flowering in autumn^35^. Sherry, *et al*.^36^ argued that plants blooming before the peak summer heat would show advanced flowering phenology, whereas plants blooming after the peak summer heat would show delayed flowering. Given that the present study was conducted from September until November, the onset of flowering of *C. hirsuta* was delayed into autumn. The delay in flowering may be due to a prolonged vegetative growth period. This result indicates that the flowering phenology of plants is probably determined by its microhabitat and/or genetic factors^27^. In addition, numerous other factors may influence *C. hirsuta* performance under climate warming^37^, such as density-dependent floral induction, growth responses to warming, or soil desiccation. In the present experiment, we precisely controlled the soil moisture content in the trays to eliminate bias from soil desiccation. Thus, warming, rather than soil desiccation, is considered as the most important factor in this study. Flowering phenology responded to both warming and population density treatments in the present experiment. Population density significantly affected the onset, end, duration, and the mean amplitude of the flowering.

Recent studies have observed positive^38^, negative^34^,^39^, or neutral^40^,^41^,^42^ effects of climate warming on seed output of plants in alpine and arctic areas, where the effects of warming are likely to be most pronounced. Here, we observed a positive effect of warming on the number of flowers and seeds produced per plant, but a negative effect on 1000-seed weight (Fig. 3) and reproductive allocation (Fig. 4), for an ephemeral herb in a subtropical area. Under the same plant density, the number of seeds per fruit did not differ significantly between the warmed and unwarmed conditions, and no difference in the impact on fruit set was observed. These results suggested that seed set by *C. hirsuta* may have been mainly limited by pollen, but not resource availability. Under the warmed condition, *C. hirsuta* showed a “mass-flowering” pattern, which favored pollination and production of both fruit and seeds because a high density of flowers was advantageous for pollen dispersal. *C. hirsuta* produced seeds with significant larger diameter and showed greater reproductive allocation in the unwarmed condition than those in the warmed condition, which indicated that an earlier flowering phenology, especially an earlier onset of flowering, may be advantageous for plants to develop mature seeds before the end of the growing season^43^. In addition, resource limitation in fruit and seed development may be another important factor.

In conclusion, our results indicated that the high plant density treatment significantly advanced the first flowering dates, shorten the duration of flowering, and decreased the production of flowers, which means population density significantly influences flowering phenology and sexual reproduction. The most likely responsible mechanism for this phenomenon may be resource limitation to sexual reproduction. It was reported that competition for resources is primary to original plant species in a given constant density^44^. At high plant densities, the conditions were unfavorable to sexual reproduction because of crowding and competition for resources.

## Materials and Methods

### Plant material

*Cardamine hirsuta* L. (Brassicaceae) is an ephemeral edible herb which begins to flower in one month after seed germination and the life cycle is less than three months. It lasted 4 to 10 days for the herb to flower and spread the seeds. And the seeds can germinate within a few days after dispersed. In the present study, the seeds with the same inherited characteristic of the herb were purchased from the seed company, and sowed in different densities as described in “Experiment design”.

We examined the effect of simulated warming on flowering phenology and sexual reproduction under the four plant densities. The main indicators of sexual reproduction included the length and diameter of fruit, the numbers of fruit per plant, and the numbers of seeds per fruit, and 1000-seed weight which are key indexes to reflect the characteristics of plant sexual reproduction. During the flowering period, we observed the flowering course of each individual plant, including such indexes as initial time of flowering (or first flowering dates), last flowering dates (or the final one), numbers of flower per plant and duration of flowering. We also counted the numbers of fruits per plant and seeds per fruit, measured the lengths and diameters of the fruits and weighed the thousand-seed weight after the seed mature. At the end of the the experiment, each part of the plants was harvested and oven dried at 80 °C for 48 h and then weighed as the biomass.

### Experiment design

The experiments were conducted at the biological experiment station of Jinggangshan University, China (27°06′31′′–27°07′23′′N, 115°01′08′′–115°02′05′′E), and lasted from September to November, 2014. The seeds of *Cardamine hirsuta* were sown evenly in propagation trays (60 cm × 40 cm × 20 cm for length × width × height). After germination of the seeds, the final plant densities were adjusted according to the growth densities in field to 600, 2000, 4000 and 10,000 stems m^−2^ in each four trays. All of the plants were grown outdoors without water or nutrient limitation.

**Figure 5:**
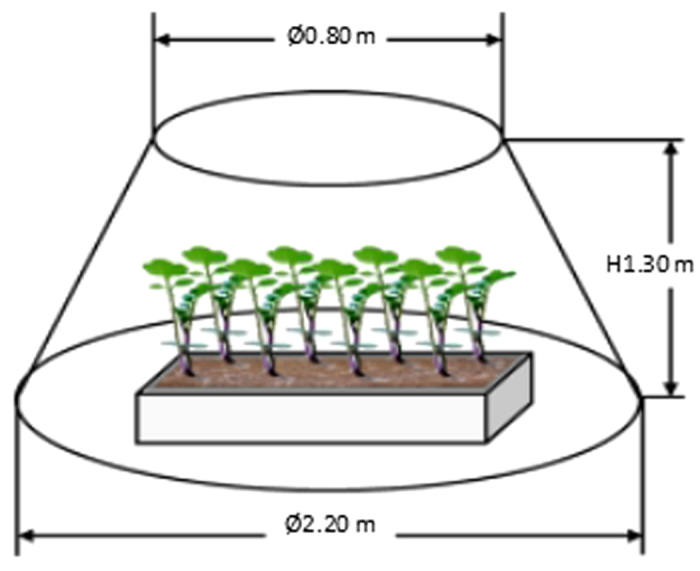
Schematic illustration of the open-top chambers used in the experiments.

To examine the effect of climate warming on flowering phenology and sexual reproduction, 5 conical open-top chambers (OTCs) with 2.20 m basal diameter, 0.80 m top diameter, and 1.30 m in height were designed to simulate climate warming (Fig. 5). In each OTC, four sowing trays growing plants under different densities were randomly placed. The same number of replicate trays which didn’t enclose in open-top chambers was set as controls (CK).

**Figure 6:**
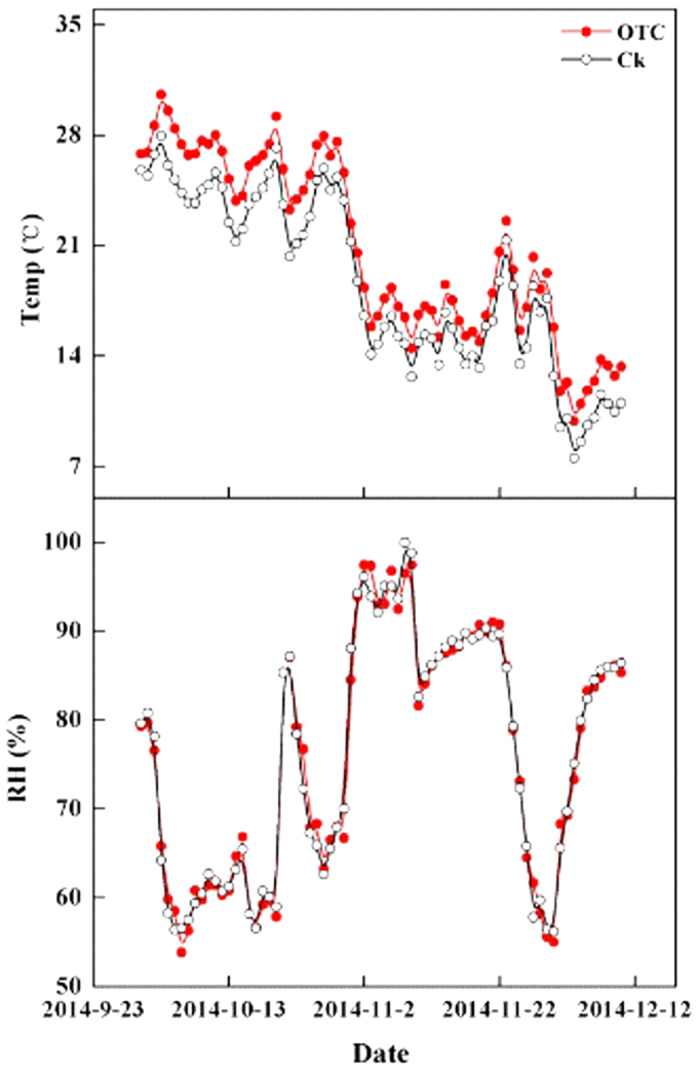
Daily average temperature and relative humidity (RH) during the study period. OTC, Open-top chamber; CK, control.

During the whole experiment, the temperature and relative humidity of the atmosphere 10 cm above the soil inside and outside the OTCs were recorded at hourly intervals with an automatic temperature and humidity recorder (EM50, Decagon Devices Inc., Pullman, USA), and then converted to the daily average temperature and relative humidity. To avoid the effect of soil moisture on the result, the soil relative humidity was controlled by watering. The results of the early experiment showed that the daily average air temperature significantly increased (*t*_963_ = 18.242, *P* < 0.0001), varying from 1.03 °C to 5.08 °C with a mean of 2.53 °C (Fig. 6), and the daily average relative humidity slightly decreased (*t*_963_ = 0.169, *P* = 0.866), varying from −3.55% to 4.5% with a mean of 0.04%. The results indicated that the amplitude of warming was consistent with the effect of OTCs in the previous studies and that the variation in relative humidity inside and outside the OTCs was not significant.

### Statistical analysis

The mutual effects of density, warming (temperature) and their interaction (density × temperature) were analysed using one-way analysis of variance (ANOVA), and the significance of difference between different treatments were comparatively analysed with the least significant difference (LSD). For flowering phenology curves, days were numbered, taking October 30 as the first day, October 31 as the second day, and so on. The analyses were performed with SPSS 18.0 (SPSS, Inc., Chicago, IL, USA) and OriginPro 8.0 (OriginLab, Northampton, MA, USA). All *p*-values were considered significant at the 0.05 significance level.

## Additional Information

**How to cite this article**: Cao, Y. *et al*. Simulated warming shifts the flowering phenology and sexual reproduction of *Cardamine hirsuta* under different Planting densities. *Sci. Rep.* **6**, 27835; doi: 10.1038/srep27835 (2016).

## Figures and Tables

**Table 1:** The analysis of variances in flowering phenology and fruiting traits of *Cardamine hirsuta* for the factors Density and Temperature.

Source	Onset					Endset				
	Sum of Squares	d.f.	Mean Square	*F*	*P*	Sum of Squares	d.f.	Mean Square	*F*	*P*
Corrected Model	1005.067^a^	7	143.581	6.911	<0.0001	939.925^a^	7	134.275	9.942	<0.0001
	17376.133	1	17376.13	836.396	<0.0001	71394.408	1	71394.41	5286.144	<0.0001
Density	264.6	3	88.2	4.245	0.007	802.025	3	267.342	19.794	<0.0001
Temperature	410.7	1	410.7	19.769	<0.0001	27.075	1	27.075	2.005	0.1596
Density × Temperature	329.767	3	109.922	5.291	0.0019	110.825	3	36.942	2.735	0.047
**a. *R*^2^ = 0.302 (Adjusted *R*^2^ = 0.258)**						**a. *R*^2^ = 0.383 (Adjusted *R*^2^ = 0.345)**				
**Duration**						**Flowers per Plant**				
Corrected Model	2547.858^a^	7	363.98	41.023	<0.0001	7666.800^a^	7	1095.257	41.26	<0.0001
	21413.408	1	21413.41	2413.426	<0.0001	15962.133	1	15962.13	601.318	<0.0001
Density	1762.092	3	587.364	66.2	<0.0001	132.3	1	132.3	4.984	0.0281
Temperature	648.675	1	648.675	73.11	<0.0001	7365.8	3	2455.267	92.494	<0.0001
Density × Temperature	137.092	3	45.697	5.15	0.0023	168.7	3	56.233	2.118	0.1017
**a. *R*^2^ = 0.719 (Adjusted *R*^2^ = 0.702)**						**a. *R*^2^ = 0.721 (Adjusted *R*^2^ = 0.703)**				
**Fruits per Plant**						**Seeds per Fruit**				
Corrected Model	7666.800^a^	7	1095.257	41.288	<0.0001	1918.125^a^	7	274.018	22.037	<0.0001
	15962.133	1	15962.13	601.723	<0.0001	92130.208	1	92130.21	7409.227	<0.0001
Density	132.3	1	132.3	4.987	<0.0275	414.408	1	414.408	33.327	<0.0275
Temperature	7365.8	3	2455.267	92.556	<0.0001	1480.225	3	493.408	39.681	<0.0001
Density × Temperature	168.7	3	56.233	2.12	0.1017	23.492	3	7.831	0.63	0.5973
**a. *R*^2^ = 0.721 (Adjusted *R*^2^ = 0.703)**						**a. *R*^2^ = 0.579 (Adjusted *R*^2^ = 0.553)**				
**Fruit Length**						**Fruit Diameter**				
Corrected Model	716.099^a^	7	102.3	50.502	<0.0001	0.498^a^	7	0.071	6.445	<0.0001
	34875.435	1	34875.44	17216.98	<0.0001	164.83	1	164.83	14921.89	<0.0001
Density	311.503	1	311.503	153.78	<0.0001	0.048	1	0.048	4.345	<0.0393
Temperature	375.911	3	125.304	61.859	<0.0001	0.43	3	0.143	12.988	<0.0001
Density × Temperature	28.685	3	9.562	4.72	0.0039	0.02	3	0.007	0.602	0.6151
**a. *R*^2^ = 0.759 (Adjusted *R*^2^ = 0.744)**						**a. *R*^2^ = 0.287 (Adjusted *R*^2^ = 0.243)**				
